# Association of SARS-CoV-2 Vaccinations with SARS-CoV-2 Infections, ICU Admissions and Deaths in Greece

**DOI:** 10.3390/vaccines10020337

**Published:** 2022-02-21

**Authors:** Foteini Malli, Ioannis C. Lampropoulos, Dimitrios Papagiannis, Ioanna V. Papathanasiou, Zoe Daniil, Konstantinos I. Gourgoulianis

**Affiliations:** 1 Respiratory Disorders Laboratory, Faculty of Nursing, University of Thessaly, 41500 Larissa, Greece; 2 Respiratory Medicine Department, Faculty of Medicine, University of Thessaly, 41500 Larissa, Greece; i.ch.lampropoulos@gmail.com (I.C.L.); zdaniil@med.uth.gr (Z.D.); kgourg@med.uth.gr (K.I.G.); 3 Public Health & Vaccines Laboratory, School of Health Sciences, Faculty of Nursing, University of Thessaly, 41110 Larissa, Greece; dpapajohn@uth.gr; 4 Community Nursing Laboratory, Faculty of Nursing, University of Thessaly, 41500 Larissa, Greece; iopapathanasiou@uth.gr

**Keywords:** COVID-19, SARS-CoV-2, vaccination

## Abstract

The available coronavirus disease 2019 (COVID-19) vaccines have shown their effectiveness in clinical trials. We aimed to assess the real-world effects of SARS-CoV-2 vaccinations in Greece. We combined national data on vaccinations, SARS-CoV-2 cases, COVID-19-related ICU admissions and COVID-19-related deaths. We observed 3,367,673 vaccinations (30.68% of the Greek population), 278,821 SARS-CoV-2 infections and 7401 COVID-19-related deaths. The vaccination rate significantly increased from week 2 to week 6 by 85.70%, and from week 7 to 25 by 15.65%. The weekly mean of SARS-CoV-2 cases, COVID-19 ICU patients and COVID-19 deaths markedly declined as vaccination coverage accumulated. The rate of SARS-CoV-2 cases increased significantly from week 2 to week 13 by 16.15%, while from weeks 14–25 the rate decreased significantly by 13.50%. The rate of COVID-19-related ICU admissions decreased significantly by 7.41% from week 2 to week 4, increased significantly by 17.22% from weeks 5–11, then decreased significantly from weeks 17–20, by 11.99%, and from weeks 21–25, by 16.77%. The rate of COVID-19-related deaths increased significantly from week 2 to week 15 by 12.08% and decreased significantly by 16.58% from weeks 16–25. The data from this nationwide observational study underline the beneficial impact of the national vaccination campaign in Greece, which may offer control of the SARS-CoV-2 pandemic.

## 1. Introduction

Coronavirus Disease 19 (COVID-19) has emerged as an ongoing pandemic that has resulted in more than 5 million deaths worldwide [1]. The fatality ratio of COVID-19 cases (i.e., the number of deaths divided by the number of diagnoses) ranges by region from 19.48% to below 1% [2]. Mortality is increased in elderly patients, males and subjects with comorbidities such as diabetes, arterial hypertension and cardiovascular disease [3]. 

In response to the pandemic, several attempts have been made to control the dissemination of the SARS-CoV-2 infection in order to reduce COVID-19-related morbidity and mortality. Recently, authorization of COVID-19 vaccines took place, soon after the publication of the initial phase 3 trials [4], and to date more than 8 billion vaccine doses have been administered worldwide [2]. In randomized clinical trials, their rates of effectiveness against symptomatic COVID-19 were 95% for the BNT162b2 vaccine (Pfizer BioNTech), 70.4% for the ChAdOx1 nCoV-19 vaccine (AZD1222, Astra Zeneca) and 94.1% for the mRNA-1273 SARS-CoV-2 vaccine (Moderna) [5,6,7]. The emergency development of COVID-19 vaccines, as well as beliefs that these vaccines are not effective, have led to negative attitudes and vaccine hesitancy worldwide [8,9]. Large epidemiological studies are warranted in order to estimate the real-world effectiveness of COVID-19 vaccines in order to overcome the aforementioned barriers to vaccine acceptance.

The first case of COVID-19 in Greece was observed on the 26 February 2020 [10]. The vaccination campaign in Greece began on the 4 January 2021, during the second pandemic wave [11]. A third pandemic wave occurred in Greece during March 2021 and the total COVID-19 cases exceeded 200,000 [12]. By the end of May 2021, Greece recorded a total of 400,000 COVID-19 cases and entered the resolution phase of the third wave. The temporal impact of the vaccination campaign in the course of the pandemic in Greece has not been reported previously.

In the present study we aim to address the effectiveness estimates for COVID-19 vaccinations in the first 25 weeks of the national vaccination campaign in Greece. To this end, we associated the rate of vaccinations against SARS-CoV-2 with the rate of new SARS-CoV-2 cases, COVID-19-related ICU admissions and COVID-19-related deaths.

## 2. Materials and Methods

### 2.1. Study Population

We used national surveillance data to address the association of SARS-CoV-2 vaccination on COVID-19 outcomes (i.e., new SARS-CoV-2 cases, COVID-19-related ICU admissions and COVID-19-related deaths). We analyzed data from the 1st to the 25th week of the vaccination campaign. Data concerning vaccinations were retrieved from the European Centre for Disease Prevention and Control (ECDC) [13]. Vaccination trend analysis included data from fully completed vaccinations. Data about COVID-19 infections and outcomes were retrieved from the National Public Health Organization (NPHO) of Greece [14]. 

The nationwide vaccination campaign in Greece began on the 4 January 2021. The vaccination was initially applied to health care workers (1st week), subjects older than 85 years old (2nd week), while on the 3rd week of vaccinations, vaccine availability extended to subjects in the age group 80–84 years. At week 6, vaccination was extended to citizens of 60–64 years old and 75–79 years old. At week 13, vaccine availability was extended to ages 65–69 years, at week 16, to ages 50–59 years and at week 17, to ages 30–49 years. The vaccination program was started with the Pfizer/BioNTech vaccine and later the Oxford/Astra Zeneca, Moderna and Johnson & Johnson were subsequentially introduced. Vaccination was free of charge for the subjects vaccinated. 

The three outcomes assessing vaccination effectiveness were as follows: 1. SARS-CoV-2 cases were defined as laboratory confirmed SARS-CoV-2 cases (symptomatic or asymptomatic); 2. Severe COVID-19 patients were defined as those admitted to the ICU; 3. Deaths attributed to COVID-19 were defined as deaths in patients with confirmed COVID-19. 

The National Public Health Organization of Greece (NPHO) collects the data from all the diagnostic laboratories and reports all the daily laboratory confirmed cases of SARS-CoV-2 infections. All hospitals provided daily updates on the number of new cases, ICU admissions, deaths, etc., and the data are provided to the National database. 

ECDC data on vaccinations are reported for the following age groups: 18–24 years, 25–49 years, 50–59 years, 60–69 years, 70–79 years and ≥80 years, while NPHO reports data for the age groups 0–17 years, 18–39 years, 40–64 years and ≥65 years. In order to perform subgroup analysis of the vaccination effect according to age, we grouped the data for patients aged <69 years old in ECDC with those of patients aged ≤65 years in NPHO files while data from patients ≥70 years in ECDC and ≥65 years in NPHO were also grouped together. 

Ethics approval was not applicable since we revised publicly available national surveillance data. No identifiable demographic or personal data were used in the present study.

### 2.2. Statistical Analysis

Data are presented as absolute numbers or as percentages. We used joinpoint regression modelling in order to assess the variation in the trends in vaccination rates, SARS-CoV-2 cases, COVID-19-related ICU admissions and COVID-19-related deaths. The joinpoint regression model investigates the combinations of trends that result in a statistically significantly better fit to a data series than a single-trend line fitted using Poisson regression or time-series models [15]. With this procedure, one may determine the number of joinpoints that are sufficient for the estimation of significant alterations in incidence trends over time. The Joinpoint Regression Program (3.5.2) and SPSS 20 were used to analyze the data. A statistically significant joinpoint was set at *p* < 0.05.

## 3. Results

The first vaccination efforts in Greece started on the 27 December 2020 with sparse vaccinations, whilst the nationwide vaccination campaign began on the 4 January 2021 (week 1) with the vaccination of healthcare workers, and was extended on the 19 January to persons aged ≥ 85 years. The vaccination campaign started while Greece was under a nationwide lockdown, which had begun on the 7 November 2020. Phased recession of the restriction measures occurred on the 11 January 2021 (the 2nd week of the vaccination campaign) with the opening of school facilities.

During the study period, there were 278,821 new SARS-CoV-2 infections and 7401 COVID-19-related deaths. At the end of the study period, 3,367,673 full vaccinations occurred (19.49% in aged <70 years), which amounts to 30.68% of the Greek population. Table 1 presents the weekly distribution of vaccinations, new SARS-CoV-2 cases, ICU admissions due to COVID-19, COVID-19-related deaths and the ratio of COVID-19-related ICU admissions/SARS-CoV-2 cases and the ratio of COVID-19-related deaths/SARS-CoV-2 cases. 

**Figure 1:**
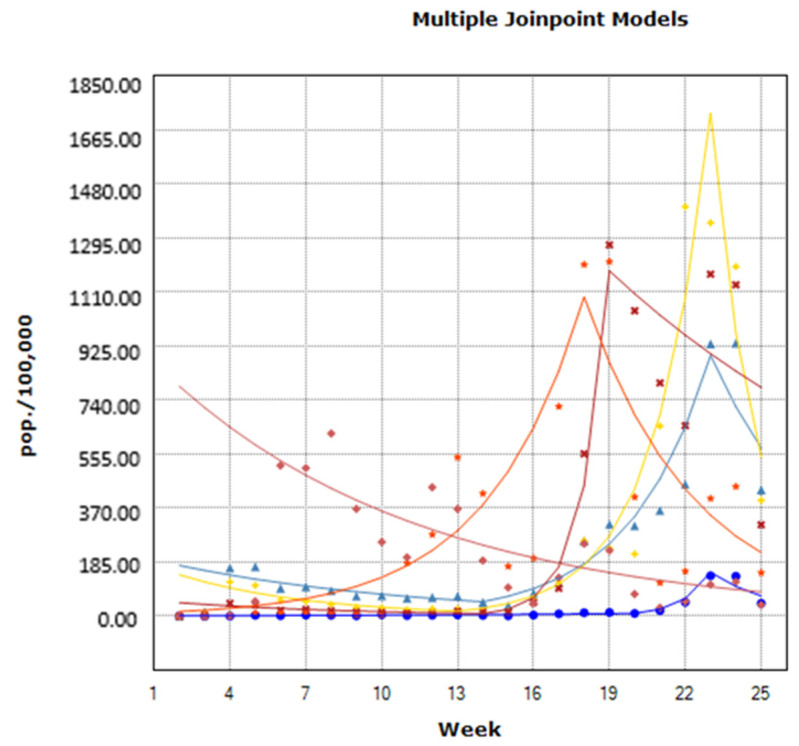
The weekly course of vaccinations per 100,000 population in Greece per age group (18–24 years: blue dots, 25–49 years: blue triangles, 50–59 years: yellow triangles, 60–69 years: red x, 70–79 years: orange asterisk, >80 years: red rhombus). The number on the y axis correspond to the number of vaccinations per 100,000 population. The X axis corresponds to weeks of vaccination.

The course of vaccinations by age group from week 1 to week 25 is presented in Figure 1. According to the results of the joinpoint analysis, for the age group of 18–24 years, there was a significant increase in the rate of vaccinations by 9.63% (Cis: 4.7–14.8) from week 2 to week 19, which was followed by a significant increase by 148.21% (Cis: 46.5–320.5) from week 20 to week 22. The rate decreased significantly by 31.96% (Cis: −51.6–−4.3) from week 23 to week 25 (Figure 1). For subjects 25–49 years, we observed a significant decrease in the rate of vaccinations by 9.91% (Cis: −17.5–−1.6) from week 2 to week 13, which was followed by a significant increase by 37.79% (Cis: 24.8–52.2) from week 14 to week 22, while during weeks 23–25 the rate of vaccinations did not significantly differ. For the age group 50–59 years, vaccination displayed a significantly decreasing trend from week 2 to week 12 by 17.29% (Cis: −30.5–−1.6). The rate significantly increased by 58.12% from week 13 to 22 (Cis: 43.3–74.4) and significantly decreased by 43.72% (Cis: −67.5–−2.6) from week 23 to 25. For the age group 60–69 years, we did not observe statistically significant changes in vaccination rates from week 2 to 13 and from week 19 to 25, while the rate significantly increased by 164.52% (Cis: 26.6–452.9) for week 14 to 18. For the persons aged 70–79 years, we observed a significant increase of 30.08% (Cis: 9.6–54.4) for the weeks 2 to 17 and a significant decrease of 20.57% (Cis: −33.1–−5.6) for the weeks 18 to 25. Finally, for persons aged ≥80 years, there was a significantly decreasing trend in the vaccination rate from week 2 to week 25 by 9.3% (Cis: −13.0–−5.4).

**Figure 2:**
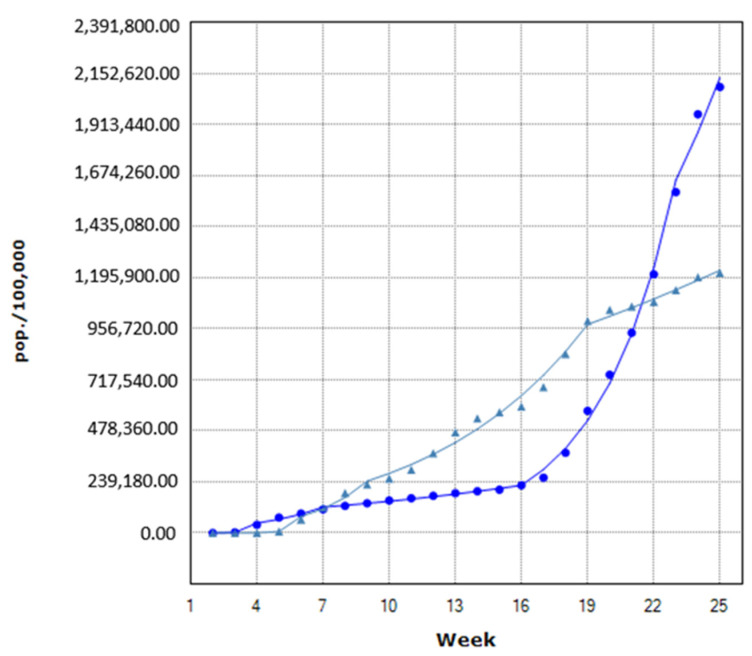
Average weekly vaccination trends by age in subjects <70 years (blue dots) and those >70 years (blue triangles).

Weekly trends in vaccinations in persons aged ≥70 years in comparison with those <70 years are presented in Figure 2. For subjects aged <70 years, we did not observe significant differences in the vaccination rate from week 2 to week 6, while for week 7 to week 15, we observed a significant increase in the vaccination rate by 7.03% (Cis: 3.8–10.4), which was followed by a significant increase of 33.07% (Cis: 29.7–36.6) for week 16 to 22 and a significant increase of 13.61% (Cis: 4.8–23.1) for week 23 to 25 (Figure 2). For subjects ≥70 years, vaccination rates were not statistically significantly different between week 2 and week 5. The vaccination rate significantly increased by 46.49% (Cis: 5–104.4) for week 6 to week 8, by 14.95% (Cis: 13–16.9) for week 9 to 18 and by 3.92% (Cis: 1.8–6) for week 19 to 25. 

**Figure 3:**
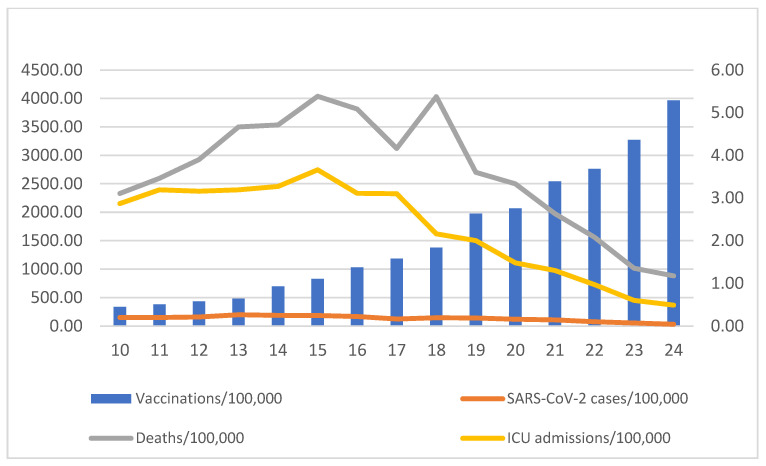
The course of vaccinations (per 100,000 population) in relation to weekly average of SARS-CoV-2 cases, COVID-19-related ICU admissions and COVID-19 deaths.

**Figure 4:**
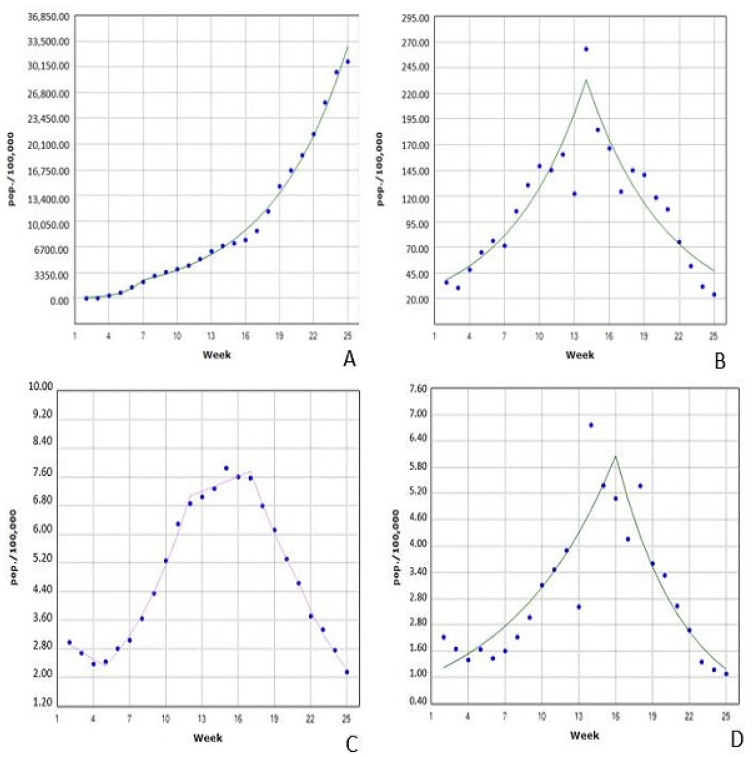
The course of vaccinations (per 100,000 population) (**A**), trends in the rate of SARS-CoV-2 cases (**B**), COVID-19-related ICU admissions (**C**) and COVID-19-related deaths (**D**) during the study period.

As vaccination coverage accumulated nationwide, we observed that the weekly mean value of SARS-CoV-2 cases, COVID-19 ICU patients and COVID-19 deaths markedly declined (Figure 3). Figure 4 presents the results of joinpoint analysis for the trends in the rate of vaccinations, SARS-CoV-2 cases and COVID-19-related ICU admissions and deaths. In more detail, for all ages, the vaccination rate significantly increased from week 2 to week 6 by 85.70% (Cis: 28.1–169.1), and from week 7 to week 25 by 15.65% (Cis: 14.9–16.4). The rate of SARS-CoV-2 cases increased significantly from week 2 to week 13 by 16.15% (Cis: 11.1–21.5), while from week 14 to week 25 the rate decreased significantly by 13.50% (Cis: −17.7–−9.1). For the whole study duration, the rate of SARS-CoV-2 cases remained stable with a marginal difference of 0.9% (Cis: −2.2–4.4). For ICU admissions related to COVID-19, the rate decreased significantly by 7.41% (Cis: −13.5–−0.9) from week 2 to week 4, followed by a significant increase of 17.22% (Cis: 15.1–19.4) from week 5 to week 11. The rate of ICU admissions remained stable during week 12 to week 16 (Cis: −0.7–4.6). The ICU admissions rate decreased significantly from week 17 to week 20 by 11.99% (Cis: −15.9–−7.9) and from week 21 to week 25 by 16.77% (Cis: −20.2–−13.2). For the whole study period, the rate of ICU admissions displayed a small reduction by 1.2% (−2.6–0.2), which did not reach statistical significance. Finally, the rate of COVID-19-related deaths increased significantly from week 2 to week 15 by 12.08% (Cis: 8.5–15.8) and decreased significantly by 16.58% (Cis: −22.4–−10.4) from week 16 to week 25. 

**Figure 5:**
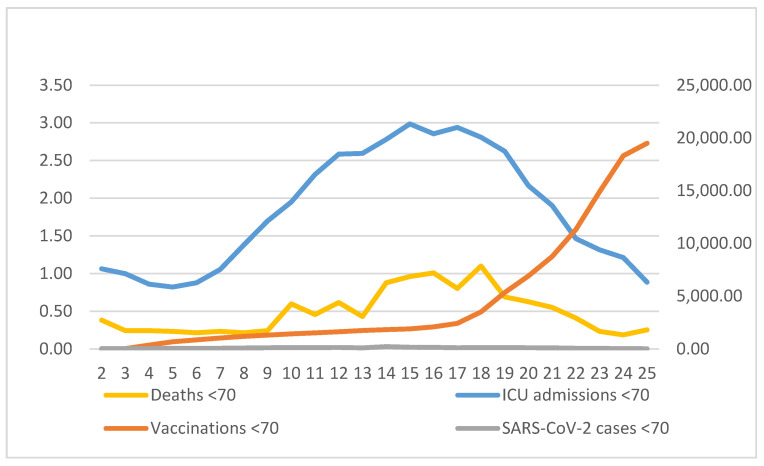
Average of vaccinations, weekly SARS-CoV-2 cases, ICU admissions and deaths for subjects <70 years. Data are expressed per 100,000 population. The left y axis corresponds to ICU admissions and deaths. The right y axis refers to vaccinations and SARS-CoV-2 cases.

Figure 5 presents the average of vaccinations, weekly SARS-CoV-2 cases, ICU admissions and deaths for subjects <70 years. In this age group, the rate of SARS-CoV-2 cases increased statistically significantly by 16.18% from week 2 to week 13 (Cis: 10.8–21.8) followed by a statistically significant decrease of 12.59% (Cis: −16.9–−8.1) from week 14 to week 25. ICU admissions decreased statistically significantly by 10.53% (Cis: −18.4–−1.9) from week 2 to week 4, which was followed by a statistically significant increase of 20.39% (Cis:16.2–24.7) from week 5 to week 10 and a statistically significant increase of 6.1% (Cis: 0.5–12.1) from week 11 to week 14. For weeks 15 to 18, we did not observe a significant change in SARS-CoV-2 ICU admissions but there was a decrease of 16.09% between week 19 and the end of the study period. For COVID-19 deaths, we observed a statistically significant increase by 10.64% (Cis: 7.2–14.2) from week 2 to week 17, which was followed by a statistically significant decrease by 22.03% (Cis: −31–−11.9) from week 18 to week 25. 

**Figure 6:**
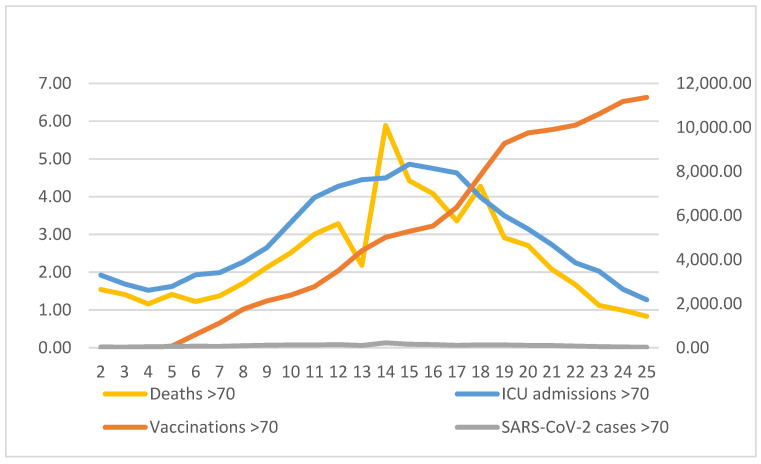
Average of vaccinations, weekly SARS-CoV-2 cases, ICU admissions and deaths for subjects >70 years. Data are expressed per 100,000 population. The left y axis corresponds to ICU admissions and deaths. The right y axis refers to vaccinations and SARS-CoV-2 cases.

Figure 6 displays the average of vaccinations, weekly COVID-19 cases, ICU admissions and deaths for subjects ≥70 years. For SARS-CoV-2 cases, we observed an increase by 16.23% (Cis: 12.1–20.5, *p* < 0.05) from week 2 to week 13 while cases decreased significantly by 19.41% (Cis: −23.4–−15.2) from week 14 to week 25. For ICU admissions, we did not observe significant changes from week 2 to week 4, but cases increased significantly by 16.29% (Cis: 13.3–19.4) form week 5 to week 11 and decreased by 14.3% (Cis: −15.8–−12.7, p < 0.05) from week 19 to week 25. From week 12 to week 14 and from week 15 to 18 they remained rather stable. For COVID-19 deaths, we observed a statistical increase by 11.9% (Cis: 8.3–15.6) from week 2 to week 15, followed by a statistically significant decrease by 17.1% (Cis: −22.9–−10.8) from week 16 to week 25. Overall (for the whole study period), in subjects ≥70 we observed a decrease in new SARS-CoV-2 cases by 2.4% (Cis: −5.2–−0.4), a decrease in ICU admissions by 1.2 (Cis: −2.8–−0.4) and a decrease in COVID-19 deaths by 0.5 (Cis: −3.7–−2.8). 

Additionally, we analyzed COVID-19-related ICU admissions and death trends according to age and gender status. For COVID-19-related ICU admissions in the female population, for subjects 18–39 years old, we observed an increase by 143.9% (Cis: 25.5–374.0) from week 2 to week 4 and a further significant increase by 25.9% (Cis: 6.5–47.7) from week 8 to week 13. For weeks 14–25, we observed a significant decrease by 12.8% (Cis: −17.7–−7.7). The rate remained stable for weeks 5–7. For females aged 40–64 years old, we observed a significant increase in COVID-19-related ICU admissions by 8.0% (Cis: 4.2–11.9) for weeks 2–16, which was followed by a significant decrease by 13.7% (Cis: −21.4–−5.2) for weeks 17–25. For females older than 65 years old, we observed a significant increase in ICU admissions by 10.8% (Cis: 8.1–13.5) for weeks 2–15 and a significant decrease by 13.6% (Cis: −17.7–9.3) for weeks 16–25. For COVID-19-related ICU admissions in the male population aged 18–39 years, we observed a significant decrease by 15.7% (Cis: −26.5–3.2) for weeks 17–25. For this age group, for weeks 2–16, we recorded a not significant increase by 4.1% in ICU admissions. For males aged 40–64 years, we observed a not significant increase by 7.2% for weeks 2–16, which was followed by a significant decrease by 13.8% (Cis: −19–−8.4) for weeks 17–25. Finally, for males older than 65 years, we observed a significant increase of ICU admissions by 20.2% (Cis: 8.1–33.6) for weeks 7–10, which was followed by stability in the rate of admissions until week 16. For weeks 17–25, we recorded a significant decrease by 13.5% (Cis: −15.8–−11.2).

For COVID-19-related deaths, in the female population aged 18–39 years, we did not observe significant differences during the study period. For female subjects 40–64 years, deaths were rather stable for weeks 2–5 while deaths increased by 21.0% (Cis: 8.9–34.5) for week 6 to week 15. For week 16 to week 25, we observed a significant decrease by 15.5% (Cis: −23.2–−7.1). For females older than 65 years, we observed stability of the death rate for week 2 to week 5. For week 6 to week 14 we observed a significant increase by 17.2% (Cis: 10.3–4.6), which was followed by a significant decrease of 14.6% (Cis: −18.6–−10.3) for weeks 15–25. In the male population, deaths for ages 18–39 years were not recorded during the study period. For ages 40–64 years, we observed a significant increase of 10.0% (Cis: 6.4–13.7) for weeks 2–16, which was followed by a decrease of 17.3% (Cis: −24.8–−9.0) for weeks 17–25. For males older than 65 years, we did not observe significant differences from week 2 to week 16, while for week 17 to week 25 we recorded a significant decrease in the death rate of 19.4% (Cis: −27.1–−10.9). 

## 4. Discussion

The data from this nationwide observational study underline the beneficial impact of the national vaccination campaign in Greece. As the vaccinations accumulated, we observed a significant decrease in SARS-CoV-2 cases, ICU admissions and deaths. The decreases were evident sooner in subjects ≥70 years, who were vaccinated earlier than younger subjects. The declines in COVID-19 cases and outcomes occurred despite the fact that Greece was in a phase of reopening (following a national lockdown) since the 2nd week of the vaccination campaign.

The COVID-19 vaccines have shown effectiveness against the disease in randomized clinical trials and are now widely used in national vaccination campaigns worldwide. The BNT162b2 vaccine (Pfizer BioNTech) has 95% efficacy against COVID-19 [5]. Vaccine effectiveness against symptomatic COVID-19 was estimated at 70.4% for the ChAdOx1 nCoV-19 vaccine (AZD1222, Astra Zeneca) [7] and 94.1% for the mRNA-1273 SARS-CoV-2 vaccine (Moderna) [6]. Although randomized clinical trials provide important information regarding vaccine effectiveness, their population may have differences from the general population and thus it is essential to examine and report real-world effectiveness. Fukutami et al. [16] have used a public access COVID-19 database alongside a cases, vaccinations and COVID-19 (CaVaCo) tool to assess the efficiency of SARS-CoV-2 vaccination worldwide. The authors reported heterogeneity in the effects of vaccination across countries, with the majority of them exhibiting a positive correlation between COVID-19 vaccination, new SARS-CoV-2 cases and COVID-19 deaths. In Greece, we observed that as cumulative vaccination coverage increased, the weekly average incidence of SARS-CoV-2 cases and COVID-19 deaths decreased, after the 16th week of vaccination, despite the fact that the country was undertaking a phased reopening (that started at the 2nd week of the vaccination campaign) following the nationwide lockdown. Our national population-level study adds important data on the effect of the vaccination campaign among the Greek population and provides insights into the real-life effects of vaccines in reducing the rates of SARS-CoV-2 cases and severe COVID-19 outcomes (ICU admissions and deaths). 

We observed marked declines in SARS-CoV-2 incidence (as suggested by new SARS-CoV-2 cases) and COVID-19 outcomes (i.e., ICU admissions and deaths) corresponding to increased vaccine uptake by the general population. Similar observations have been reported by Haas et al. in Israel [17], where COVID-19 vaccination proved effective in reducing symptomatic and asymptomatic SARS-CoV-2 infections, COVID-19-related severe and non-severe hospitalizations and COVID-19-related deaths. In a large community surveillance study in England, COVID-19 vaccination resulted in reductions in SARS-CoV-2 infections of 79% after the ChAdOx1 vaccine and of 80% after the BNT162b2 vaccine [18]. Similarly, in a retrospective study performed in an Italian province, the effectiveness of COVID-19 vaccination was estimated at 95% for the prevention of SARS-CoV-2 infections or COVID-19-related deaths [19]. In the same context, the real-world effectiveness of the vaccines against symptomatic disease or severe COVID-19 has been reported in elderly subjects [20]. Our results are in accordance with the aforementioned findings and provide further support for the impact of COVID-19 vaccination. In our study, the reductions in SARS-CoV-2 cases, ICU admissions and deaths occurred despite the fact that the country was in a phased reopening following the implementation of a nationwide lockdown of approximately 2 months’ duration. Importantly, the COVID-19-related outcomes remained low even after the reopening had occurred, suggesting a positive impact of the COVID-19 vaccination campaign on public health. 

Herd immunity occurs when a large percentage of the population is immune, resulting in decreased spread of the disease from person to person and thus protection of the whole community rather than immune subjects only. Historically, herd immunity was thought to be reached when approximately 65–70% of the population has been immunized [21]. We observed a declining trend in new SARS-CoV-2 cases starting in the 14th week of the vaccination campaign when approximately 6.1% of the population was vaccinated. One study has reported that for some countries the infection rate after the vaccination campaign has an inverted U-shaped trend that is characterized by an increasing rate of infection after vaccination starts, which reaches a peak then declines as vaccinations accumulate [21]. Our results are in accordance with the aforementioned study and suggest that in some countries, presumably those that are underpopulated, like Greece, partial herd immunity may be reached earlier, and that the nationwide vaccination campaign should be intensive so as to quickly reach the turning point and prevent SARS-CoV-2 resurgence. The emergence of SARS-CoV-2 variants with increased transmissibility may lead to higher herd immunity thresholds, and efforts should be made to increase vaccine uptake in order to reduce SARS-CoV-2 transmission and morbidity [17]. 

Vaccine hesitancy results in a delay or refusal of vaccination despite vaccine availability and, in the COVID-19 era, it has emerged as a growing global threat to public health [22]. Although some populations, such as health care workers, have shown high acceptance of COVID-19 vaccination, other groups are more hesitant [23,24]. Safety concerns, doubts about the efficacy of the available vaccines and misinformation about the virus are some of the reasons underlying COVID-19 vaccine hesitancy, which may result in slower vaccination rates [25]. A study that was conducted in the USA presents statistically significant differences in vaccine hesitancy based on sociodemographic characteristics, with the highest prevalence of COVID-19 vaccine hesitancy found among African Americans, Hispanics, those who had children at home, individuals with lower education and incomes and rural dwellers [26].

Our study is not without limitations. We report reduced rates of SARS-CoV-2 cases, ICU admissions and deaths coinciding with the accumulation of vaccinations, but one cannot rule out the effect of potential cofounders and the positive contribution of other factors. One may consider the analysis of national surveillance data a limitation. The analysis of publicly available data is a common research method that may help answer research questions concerning global (or, in our case, national) responses to the novel coronavirus. The ecological design of our study cannot discriminate the impact of non-pharmaceutical interventions; however, it is notable that despite the reopening of the national lockdown we observed a significant drop in SARS-CoV-2 cases, ICU admissions and deaths as the vaccinations accumulated. Unfortunately, we do not have data on the vaccination status of, or the vaccines administered to, the patients with COVID-19, which could provide direct data regarding the effectiveness of the vaccines. Additionally, we do not have available demographic characteristics (age and gender) of the COVID-19 patients or the vaccinated subjects. Therefore, we cannot perform multivariate analysis to test for the possible effects of gender and age on COVID-19-related outcomes. We must also acknowledge that a significant limitation of the present study is the fact that the comparison of the trend of vaccinations with COVID-19 outcomes according to age relied on the grouping of age groups that were not identical, due to differences in reporting of data between the NPHO and ECDC that might have resulted in misclassification. 

## 5. Conclusions

In conclusion, we observed a temporal association between vaccine uptake and reductions in the rate of SARS-CoV-2 cases, COVID-19-related ICU admissions and COVID-19-related deaths. Our data suggest that high vaccine uptake may represent an efficient route towards normality, offering control of the SARS-CoV-2 pandemic as well as reducing the morbidity and mortality of COVID-19.

## Figures and Tables

**Table 1:** Weekly distribution of vaccinations, new SARS-CoV-2 cases, ICU admissions due to COVID-19, COVID-19-related deaths and the ratio of COVID-19-related ICU admissions/SARS-CoV-2 cases and the ratio of COVID-19-related deaths/SARS-CoV-2 cases. The data on vaccinations are presented cumulatively. The data are presented as absolute numbers.

Week	Vaccinations			New SARS CoV-2 Cases Per Week			ICU Admissions Per Week			Deaths Per Week			COVID-19-Related ICU Admissions/SARS-CoV-2 Cases			COVID-19-Related Death/SARS-CoV-2 Cases		
	All Ages	<70	>70	All Ages	<70	>70	All Ages	<70	>70	All Ages	<70	>70	All Ages	<70	>70	All Ages	<70	>70
2	0	0	0	3852	3121	731	320	114	206	206	41	165	0.083	0.037	0.282	0.053	0.013	0.226
3	3140	3127	13	3273	2664	609	288	107	181	177	26	151	0.088	0.040	0.297	0.054	0.010	0.248
4	38,928	38,395	533	5172	4243	929	255	92	163	150	26	124	0.049	0.022	0.175	0.029	0.006	0.133
5	80,021	72,725	7296	6984	5820	1164	262	88	174	176	25	151	0.038	0.015	0.149	0.025	0.004	0.130
6	155,302	91,416	63,886	8178	6939	1239	301	94	207	154	23	131	0.037	0.014	0.167	0.019	0.003	0.106
7	230,264	110,793	119,471	7677	6401	1276	326	113	213	172	25	147	0.042	0.018	0.167	0.022	0.004	0.115
8	314,397	127,079	187,318	11,275	9496	1779	391	148	243	206	23	183	0.035	0.016	0.137	0.018	0.002	0.103
9	366,858	139,461	227,397	14,003	11,784	2219	466	182	284	254	26	228	0.033	0.015	0.128	0.018	0.002	0.103
10	407,622	152,272	255,350	16,003	13,286	2717	564	209	355	333	64	269	0.035	0.016	0.131	0.021	0.005	0.099
11	459,436	162,840	296,596	15,568	12,917	2651	674	248	426	371	49	322	0.043	0.019	0.161	0.024	0.004	0.121
12	548,190	174,236	373,954	17,212	14,542	2670	735	277	458	418	66	352	0.043	0.019	0.172	0.024	0.005	0.132
13	658,340	186,676	471,664	13,101	10,868	2233	755	278	477	280	46	234	0.058	0.026	0.214	0.021	0.004	0.105
14	732,985	195,776	537,209	28,219	23,557	4662	780	298	482	725	94	631	0.028	0.013	0.103	0.026	0.004	0.135
15	769,277	203,157	566,120	19,785	16,603	3182	841	320	521	577	103	474	0.043	0.019	0.164	0.029	0.006	0.149
16	815,906	223,510	592,396	17,840	15,035	2805	815	306	509	545	108	437	0.046	0.020	0.181	0.031	0.007	0.156
17	942,948	259,351	683,597	13,322	11,257	2065	811	315	496	446	86	360	0.061	0.028	0.240	0.033	0.008	0.174
18	1,215,201	375,853	839,348	15,551	13,388	2163	728	301	427	576	118	458	0.047	0.022	0.197	0.037	0.009	0.212
19	1,566,051	572,377	993,674	15,069	13,274	1795	656	281	375	386	74	312	0.044	0.021	0.209	0.026	0.006	0.174
20	1,787,368	741,728	1,045,640	12,694	11,428	1266	569	232	337	357	67	290	0.045	0.020	0.266	0.028	0.006	0.229
21	1,999,243	938,106	1,061,137	11,483	10,521	962	497	204	293	282	59	223	0.043	0.019	0.305	0.025	0.006	0.232
22	2,295,158	1,211,980	1,083,178	8063	7387	676	398	157	241	223	44	179	0.049	0.021	0.357	0.028	0.006	0.265
23	2,734,947	1,596,852	1,138,095	5551	5059	492	358	141	217	145	25	120	0.064	0.028	0.441	0.026	0.005	0.244
24	3,160,162	1,961,762	1,198,400	3412	3157	255	296	130	166	126	20	106	0.087	0.041	0.651	0.037	0.006	0.416
25	3,307,673	2,089,110	1,218,563	2584	2427	157	231	95	136	116	27	89	0.089	0.039	0.866	0.045	0.011	0.567

## Data Availability

Data are available upon request.
